# An Automated Assay System to Study Novel Tank Induced Anxiety

**DOI:** 10.3389/fnbeh.2019.00180

**Published:** 2019-08-08

**Authors:** Sara Haghani, Maharshee Karia, Ruey-Kuang Cheng, Ajay S. Mathuru

**Affiliations:** ^1^Yale-NUS College, Science Division, Singapore, Singapore; ^2^Faculty of Science, McGill University, Montreal, QC, Canada; ^3^Lee Kong Chian School of Medicine, Nanyang Technological University, Singapore, Singapore; ^4^Institute of Molecular and Cell Biology (IMCB), Singapore, Singapore; ^5^Department of Physiology, Yong Loo Lin School of Medicine, National University of Singapore, Singapore, Singapore

**Keywords:** zebrafish, anxiety, novel tank diving test, automation, open source (OS)

## Abstract

New environments are known to be anxiogenic initially for many animals including the zebrafish. In the zebrafish, a novel tank diving (NTD) assay for solitary fish has been used extensively to model anxiety and the effect of anxiolytics. However, studies can differ in the conditions used to perform this assay. Here, we report the development of an efficient, automated toolset and optimal conditions for effective use of this assay. Applying these tools, we found that two important variables in previous studies, the direction of illumination of the novel tank and the age of the subject fish, both influence endpoints commonly measured to assess anxiety. When tanks are illuminated from underneath, several parameters such as the time spent at the bottom of the tank, or the transitions to the top half of the tank become poor measures of acclimation to the novel environment. Older fish acclimate faster to the same settings. The size of the novel tank and the intensity of the illuminating light can also influence acclimation. Among the parameters measured, reduction in the frequency of erratic swimming (darting) is the most reliable indicator of anxiolysis. Open source pipeline for automated data acquisition and systematic analysis generated here and available to other researchers will improve accessibility and uniformity in measurements. They can also be directly applied to study other fish. As this assay is commonly used to model anxiety phenotype of neuropsychiatric ailments in zebrafish, we expect our tools will further aid comparative and meta-analyses.

## Introduction

Unfamiliar surroundings elicit a response of cautious exploration among animals. Among these, the open field test, originally introduced in 1934, Hall (1934) examines the motivational drive. The open field is often coupled with novel environment response test. In rodents, increased wall following and avoidance of the center, or thigmotaxis, has been used as a measure of anxiety in response to novel environments (Simon et al., 1994). The time taken or the latency to enter an operationally defined central area, or the total duration in that area has been used as an indicator of anxiolysis as animals acclimate to the novel environment (Prut and Belzung, 2003). Assays of similar nature have been used in birds and primates to model isolation-induced anxiety in novel environments (Simon et al., 1994; Moriarty, 1995).

An assay based on the concept of a novel environment, or a novel open tank as the equivalent of the commonly used open field test in rodents and its amenability for quantitative analysis in zebrafish was also described over a decade ago (Gerlai et al., 2000; Gerlai, 2003; Blaser and Gerlai, 2006). In the initial experiments, tanks were illuminated from the top to emulate an ethological context. The response of adult zebrafish in such a setup, that is, to stay at the bottom initially and slowly habituate to the rest of the tank, was interpreted as a precautionary antipredatory response followed by alleviation of anxiety, respectively. This interpretation was made on the basis of the observation that zebrafish swim at the surface of the tank for the most part of the day in the laboratory holding facilities (Gerlai et al., 2000). This type of behavior may be related to laboratory rearing conditions as field studies examining the vertical distribution of zebrafish and their gut content in the wild, in the floodplains of the Indian subcontinent, suggest that zebrafish likely occupy and feed uniformly throughout the depth of the water column in the day (Spence et al., 2006, 2007). It is nonetheless reasonable to interpret extended time spent at the bottom of a novel tank initially as an expression of anxiety, or a predator avoidance behavior. The vertical position changes rapidly and solitary zebrafish spend most of the time at the bottom of the tank when expressing innate fear after exposure to an alarm substance (Speedie and Gerlai, 2008; Parra et al., 2009; Wisenden, 2010; Mathuru et al., 2012; Gerlai, 2013). Innate fear and anxiety are dissociable, but are related phenomena that share circuits, physiological players, and behavioral expression in most animals examined (Adolphs and Anderson, 2018). Zebrafish are unlikely to be exceptions in this matter and bottom-dwelling may be a defensive strategy shared between the two phenomena.

Among the earliest studies that modeled isolation-induced anxiety in a novel tank and the effect of drugs in reducing this anxiety, was one using this assay to study the effects of nicotine (Levin et al., 2007). Subsequently, several others have used a version of this assay to examine the effect of substances known to be either anxiolytic, or anxiogenic in mammals, including humans, and established its use in zebrafish to study stress and anxiety (Bencan et al., 2009; Egan et al., 2009; Grossman et al., 2010). The main endpoint in these assays has been a quantification of the time spent in the bottom third of the tank or the diving response. Other parameters such as latency to transit to the top half and, the number of such transitions have also been used, but these are correlated with the initial diving phenomenon (Blaser and Gerlai, 2006). Erratic swimming or darting and immobility episodes are two other endpoints unrelated to diving that have been used as well. Treatment with many anxiolytics attenuates the measure of all these parameters, consistent with the interpretation that these anxiolytic compounds reduce anxiety in fish as they do in other animals by acting on common molecular targets (Bencan et al., 2009; Egan et al., 2009; Grossman et al., 2010). As a consequence, the novel tank diving (NTD) test has been used extensively and has become one of the two standard tests for anxiety in zebrafish (the other being scototaxis). A few among over a hundred studies that used this assay include (Bencan and Levin, 2008; Bencan et al., 2009; Egan et al., 2009; Cachat et al., 2010, 2013; Grossman et al., 2010; Sackerman et al., 2010; Khor et al., 2011; Maximino et al., 2011, 2013a,b; Parker et al., 2013; Pittman and Ichikawa, 2013; Vignet et al., 2013; Kulkarni et al., 2014; Mezzomo et al., 2016; Kalueff, 2017) and are reviewed in a meta-analysis (Kysil et al., 2017).

Surprisingly, however, in spite of such widespread use, the exact conditions used to perform the assay are still variable and not standardized between studies. Apart from the minor differences in the shape and size of the tank used as a novel environment, or the duration of the assay, a major difference is the manner in which the tank is illuminated. Tanks can be backlit while observing or video recording from the front (Bencan and Levin, 2008; Bencan et al., 2009; Pittman and Ichikawa, 2013), or lit from the top in a darkened room (Maximino et al., 2011, 2013a,b), or may be placed in ambient light (Egan et al., 2009; Cachat et al., 2010, 2013; Sackerman et al., 2010) with a light reflective surface at the bottom or at the tank’s back wall, or left undescribed (Grossman et al., 2010; Wong et al., 2010; Khor et al., 2011; Parker et al., 2013, 2014; Kulkarni et al., 2014; Mezzomo et al., 2016). Whether these differences influence the endpoints measured has not been systematically evaluated. We were specifically interested in the variable of illumination because adult zebrafish avoid lit areas in a scototaxis assay (Maximino et al., 2010; Lau et al., 2011), and groups (including our own), interested in observing the locomotion of zebrafish in 3D may consider illuminating the tank from underneath, or use a highly reflective surface at the bottom of the observation tank to improve contrast (Stewart et al., 2015; Audira et al., 2018).

Here, we tested if the direction of illumination, from the bottom or the top of the tank, influences the behavior of adult zebrafish in the NTD test. We find that illumination direction changes time spent and distance traversed in the bottom of the tank and the frequency of transitions to the top—that is, it affects most measures used to ascertain the level of anxiety in zebrafish. As previous studies used fish over a range of age (between 3–12 months old) in such experiments, we also examined responses of two age groups of fish, 3–5 months old adults and 7–9 months or older adults. Older fish responses are notably different in many parameters measured as they appear to be less sensitive to the illumination conditions, and acclimate faster. In effect, this means studies adopting one of the two illumination conditions, or something in between, and/or differing in the age group of fish studied can reach different conclusions.

To aid future studies, we also explored the impact of common variables such as the size of the arena used as a novel tank, the intensity of light illuminating the novel tank, and the total duration of assay. We find that each of these variables also impacts the conclusion. Another potential source of variation is the method of quantification and definitions used. For instance, darting or erratic swimming can be subjectively coded differently among different studies or differently among different observers. One way to improve reproducibility is to automate quantitation and to use clearly defined criteria that can be quantified. To this end, we also provide new open source tools with this manuscript that can be used to automate both the acquisition and the analysis of NTD behavior at a minimal cost. We expect these will allow more reliable and consistent phenotyping in studies using innate anxiety tests to investigate the genetics of comorbid mental disorders (Blaser and Rosemberg, 2012; Stewart et al., 2012; Kim et al., 2014; Meshalkina et al., 2018).

## Materials and Methods

### Experimental Method

Experiments were performed in accordance with the guidelines recommended by the Institutional Animal Care and Use Committee (IACUC) of the Biological Resource Center at A*STAR. Approved experimental protocols (IACUC 161110) were followed.

### Animals

One-hundred and forty AB wild type fish from two age groups (3–5 months old and 7–9 months old) with an equal number of males and females were used in the study. The fish were bred and grown in the laboratory fish facility (Institute of Molecular and Cell Biology, A*STAR) and housed in groups of 20–25 in 3-l tanks in standard conditions of the facility.

### Procedure

**Figure 1:**
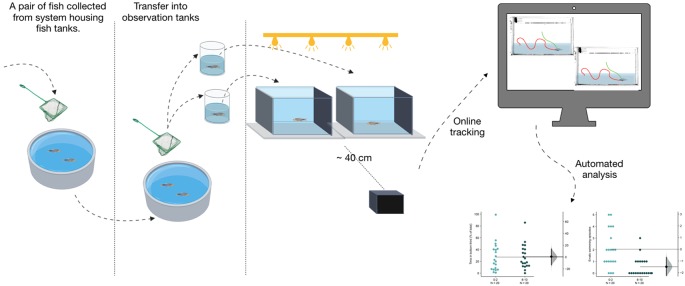
Schematic. Graphical illustration of the study method and procedure.

Prior to the experiments described, the entire procedure of the experiment including netting, transport to the behavioral observation room, and transfer into observation tanks was standardized as moving fish from their home tanks in stressful for the fish (Mathuru et al., 2017). All experiments were then conducted in the following manner (Figure 1). Fish were netted from home tanks in pairs and transferred to the behavior examination room. The netting was done using standard aquaria nets that had stitches on the sides such that the middle part of the net had no folds or obstructions. In the behavior room, fish were transferred into two separate beakers (100 ml) with ~25–30 ml of tank water immediately using the same net and were gently released into two observational glass tanks simultaneously. The transfer into beakers and release into the observation tanks were completed within 30 s. Standard observation tanks for all conditions tested were novel tanks that subject fish had not experienced. The dimensions were 20 cm × 12 cm × 5 cm; L × H × W. For the large tank condition, larger tanks of 14 cm × 12 cm × 14 cm; L × H × W were used. Tanks were filled with system water collected from the system housing the test subjects, filled up to the 10 cm mark and placed against a black background. Tank water was changed after testing four subjects.

### Illumination Method and Conditions

Tanks were uniformly illuminated in different light conditions. For low-intensity top light, a natural light LED light bar (IKEA, model LEDBERG) placed approximately 15 cm above the tanks was used to deliver a uniform illumination of 1.5 μW/mm^2^ of light when measured using a digital handheld optical power and energy meter in the 500–540 nm range (Thor Labs, PM100D). High-intensity light from the top, or the bottom was delivered using a lightbox (Artograph, LightPad 930 lx) that delivered 3 μW/mm^2^ uniform illumination in the same wavelength. The measurements made at multiple points of the observation tank showed no measurable difference in intensity. Videos were recorded using a Basler Ace (acA1300–200 μm; 1,280 × 1,024) camera placed in front of the tanks at ~40 cm distance. Videos were recorded at 10 frames per second for 600 s (10 min), except in the long duration condition where videos were recorded for 1,080 s (18 min). Subject fish could not view other fish during observation and the experimentalist were obscured by a curtain in the setup.

### Video Acquisition and Analysis

The following pipeline for data acquisition and analysis were generated for this study (Supplementary Video S1) and are provided with this article as open source Python scripts. Videos and trajectories of fish locomotion were acquired online and stored as videos, tracked videos, and an Excel file.

### Automated Tracking

The program we developed is versatile and can be adapted to any conditions, with minor modifications to the code. A detailed description is in the “Readme” file that accompanies the software at our website. In essence, the program utilizes the combination of Python and OpenCV machine vision libraries, both open source. We recommend using PyCharm community edition (also an open-source Python editor) version 5.0.2 or higher for optimal performance. The location of the fish in each image is determined by custom-written background subtraction algorithm. The background image is established by a moving average of about 10 (changeable) successive images after the fish is put into the tank. The moving average will cancel out any moving object in the image (i.e., the fish) thus resulting in a static background image. When detecting the location of the fish against the background image, a bounding rectangle of the fish is determined by using OpenCV library cv2.findContours. The center x-y coordinate of this rectangle is denoted as the center of the fish mass.

### Automated Analysis Scripts

To analyze the data generated from the automated tracker, we also developed a set of analysis scripts written in Python. These scripts are also available at our website with detailed instructions on its use described in a Readme file. These analysis scripts generate graphics as well as spreadsheets with the data, listing both absolute values and relative percentages where appropriate (for example, percentage time of total in the center of the tank, vs. along the walls). Among the parameters analyzed include, total time (in seconds), percentage of total time, average velocity, and total distance swam in the—center, along the walls, in the bottom 1/4, in the bottom 1/2, in the bottom 1/3, of the tank. Latency to make the first and second transition to the top $\frac{1}{2}$ of the tanks and the number of such transitions are also calculated. The total duration of time spent freezing (displacement of ≤ 3 mm/s) and the number of freezing episodes (at least 1 s of immobility) are also calculated by the script. Finally, the number of erratic swimming or darting episodes are also calculated. As described previously (Schirmer et al., 2013) an episode of erratic swimming was quantified as a change in instantaneous velocity that exceeded the mean swimming velocity in the period of measurement by 8 standard deviations (8 SD) or more. Importantly, though we recommend keeping all the parameters described here fixed, users can change these settings when analyzing locomotion behavior of other animals. In the context of previous experiments of NTD, our analysis scripts take into account the differences in the settings, such as the duration that a researcher may want to perform the experiment for. We recommend performing the experiment for 10 min. However, 6 min experiments performed in the past by other researchers can also be directly analyzed with our scripts. Among the two analysis scripts, one generates an output for the entire duration of the experiment and the second allows users to specify the time window of analysis (0–2 min, 5–10 min, etc.). To allow for maximum flexibility, users define inputs including the dimensions of the observation tank and the duration of the experiment during analysis. Finally, the output generated also includes 95% confidence calculations for ease of making plots and graphics independent of those generated by the script.

### Statistical Analysis

Null hypothesis significance testing and an overreliance on *p*-value based dichotomous interpretation of acceptance or rejection of a hypothesis have been criticized when studying behavior (du Prel et al., 2009; Halsey et al., 2015; Ho et al., 2019). In this manuscript, we adopted estimation statistics and Gardner-Altman plots to quantify effect sizes and to assess its precision (Ho et al., 2019). Briefly, in the figures, the primary axis (on the left) is used to represent the parameter being measured and all individual measurements are shown as a swarmplot to display the underlying distribution. Separate but aligned axes are used to show the effect size on the right, next to the groups being compared. The mean of the delta is shown by a black filled circle and the 95% bootstrap confidence intervals calculated from a nonparametric sampling of the observed data are shown by the shaded curve and whiskers. An open source website^1^ was used to generate the figures and statistical analysis presented in this manuscript. *P*-values from paired or unpaired *t*-tests as suitable were also calculated and are reported alongside confidence intervals in the following format where necessary to aid readers unfamiliar with examining effect sizes [mean difference = xyz (95% confidence intervals—upper limit, lower limit), *p* = 0.0 xyz]. The *p*-values for all the comparisons made are tabulated and plots with traditional *p*-value reporting are described in Supplementary Table S1.

## Results

### Parameters That Show an Acclimation Related Change in a Novel Tank

We used 3–5 months old adult male and female AB wild-type fish and examined their response in a novel tank when illuminated from the top, mimicking their natural habitats. Most tests of anxiety and anxiolytics consider 6-min of assay time and measure the average response over the entire duration of the experiment (Levin et al., 2007; Egan et al., 2009). We matched the conditions of the experiment described by Levin et al. (2007), who introduced the test in the form used most commonly and examined the subjects for 10-min with the expectation that fish acclimate to the novel environment over this time. We then asked which among the commonly measured parameters change consistently as a consequence of acclimation. For this purpose, we generated new open source, stand-alone, automated online tracking and analysis tools in Python to acquire the behavioral data of fish in the observation tanks (see “Materials and Methods” section).

**Figure 2:**
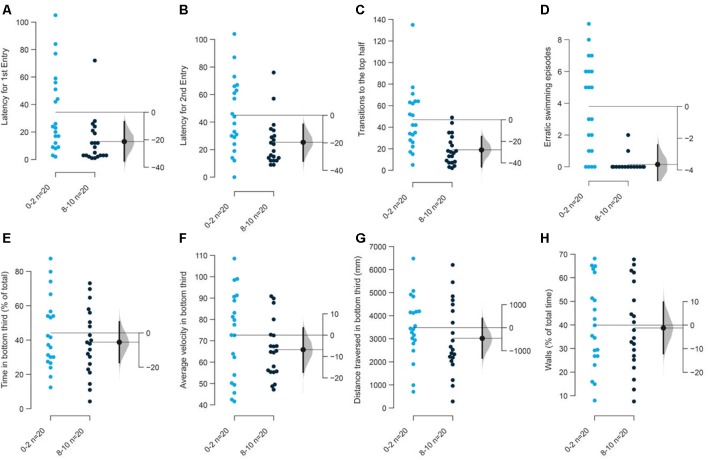
Response of 3–5 months old adult zebrafish in top lit novel tank.The first set of experiments examined the response of 3–5 months old fish in top lit tanks. The mean difference between the first 2 (0–2) and the last 2-min (8–10) of 10 min are shown in Gardner-Altman estimation plots. The latency to make the **(A)** first entry, **(B)** second entry, to the top half of the tank, the frequency of **(C)** such transitions and **(D)** of the erratic swimming episodes. The **(E)** total percentage of time, **(F)** average velocity (in mm/s), **(G)** average distance traversed (in mm) in the bottom third of the tank. **(H)** Percentage of time of the total spent swimming along the edge (thigmotaxis). Each dot in the group represents the response of one individual. *N* = 20 per group. Groups are plotted on the left axis, while the mean difference between the groups is depicted as a black dot and is plotted on a floating axis on the right. Ends of the bar around the mean shows 95% confidence interval. The shaded region in gray is 5,000 bootstrap sampling distribution.

We first tested the condition where tanks were illuminated from above uniformly at 1.5 μW/mm^2^. We found a decrease in the latency to enter the top half of the tank the first time [mean difference = −21.4 s (95 CI −35.5, −7.9), *p* = 0.006], the second time [mean difference = −19.6 s (95 CI −32.9, −4.7), *p* = 0.022], and a reduction in the number of such transitions [mean difference = −28.0 (95 CI −46.1, −15.7), *p* = 0.001], that is, factors dependent on the initial diving phenomenon are statistically significant between the first 2 vs. the last 2-min in the assay (Figures 2A–C). Fish also show fewer episodes of erratic swimming or darting [Figure 2D; mean difference = −3.65 (95 CI −4.9, −2.5), *p* = 0.0004; see “Materials and Methods” section for definition of erratic swimming episode]. On the other hand, parameters such as percentage time spent (Figure 2E), or their velocity (Figure 2F), or the distance traversed at the bottom third of the tank (Figure 2G), percentage of time following walls (Figure 2H)—other parameters measured in a novel tank assay, showed a trend but only a slight decrease only marginally. The total duration of immobility did not change in the first experiment but showed a decrease in repetition (see Figure 9).

**Figure 3:**
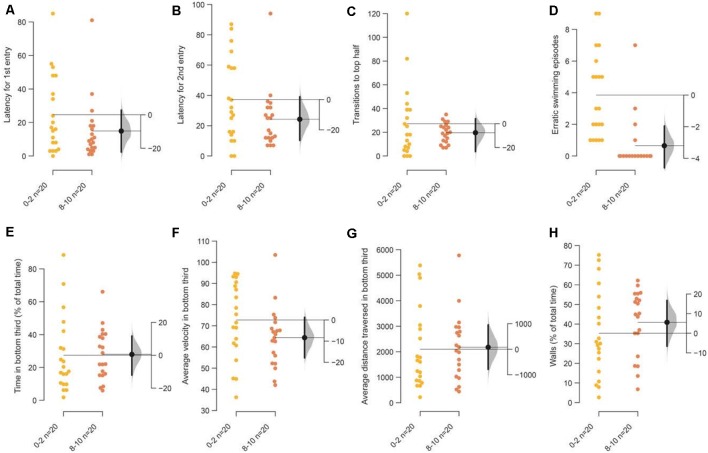
Response of 3–5 months old adult zebrafish in bottom lit novel tank. The second set of experiments examined the response of 3–5 months old fish in bottom lit tanks. The mean difference between the first 2 (0–2) and the last 2-min (8–10) of 10 min are shown in Gardner-Altman estimation plots. The latency to make the **(A)** first entry, **(B)** second entry, to the top half of the tank, the frequency of **(C)** such transitions and **(D)** of the erratic swimming episodes. The **(E)** total percentage of time, **(F)** average velocity (in mm/s), **(G)** average distance traversed (in mm) in the bottom third of the tank. **(H)** Percentage of time of the total spent swimming along the edge (thigmotaxis). Each dot in the group represents the response of one individual. *N* = 20 per group. Groups are plotted on the left axis, while the mean difference between the groups is depicted as a black dot and is plotted on a floating axis on the right. Ends of the bar around the mean shows 95% confidence interval. The shaded region in gray is 5,000 bootstrap sampling distribution.

**Figure 4:**
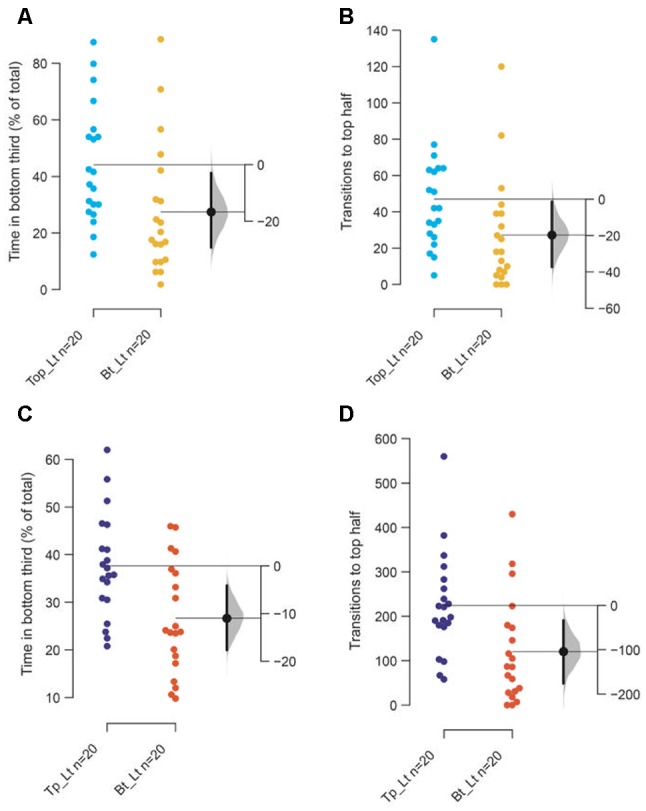
Reasons for attenuated response. The mean difference between top lit and bottom lit groups are shown in Gardner-Altman estimation plots. The **(A)** total percentage of time in the bottom third of the tank and **(B)** the frequency of transitions to the top of the tank among 3–5-month-old adults. The **(C)** total percentage of time in the bottom third of the tank and **(D)** the frequency of transitions to the top of the tank among 7–9 months or older adults. Each dot in the group represents the response of one individual. *N* = 20 per group. Groups are plotted on the left axes, while the mean difference between the groups is depicted as a black dot and is plotted on a floating axis on the right. Ends of the bar around the mean shows 95% confidence interval. The shaded region in gray is the 5,000 bootstrap sampling distribution.

**Figure 5:**
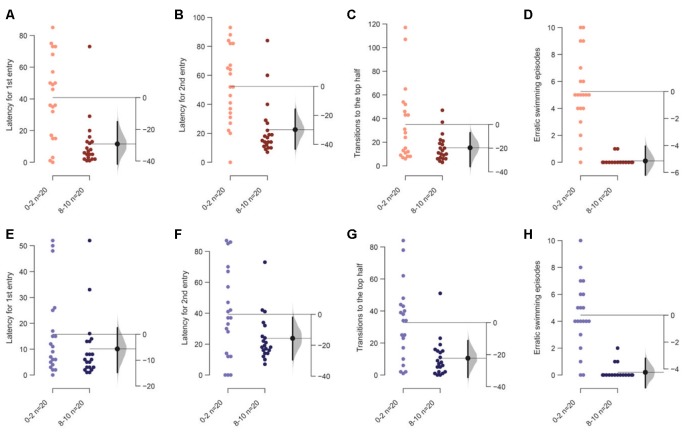
Response of 7–9 months or older adult zebrafish in a novel tank. The mean difference between the first 2 (0–2) and the last 2-min (8–10) when tanks are bottom lit or top lit are shown in Gardner-Altman estimation plots. The latency to make the **(A)** first entry, **(B)** second entry, to the top half of the tank, the frequency of **(C)** such transitions and **(D)** of the erratic swimming episodes in bottom lit condition. The mean difference between the first 2 and the last 2-min when tanks are top lit are shown in Gardner-Altman estimation plots. The latency to make the **(E)** first entry, **(F)** second entry, to the top half of the tank, the frequency of **(G)** such transitions and **(H)** of the erratic swimming episodes. Each dot in the group represents the response of one individual. *N* = 20 per group. Groups are plotted on the left axis, while the mean difference between the groups is depicted as a black dot and is plotted on a floating axis on the right. Ends of the bar around the mean shows 95% confidence interval. The shaded region in gray is 5,000 bootstrap sampling distribution.

**Figure 6:**
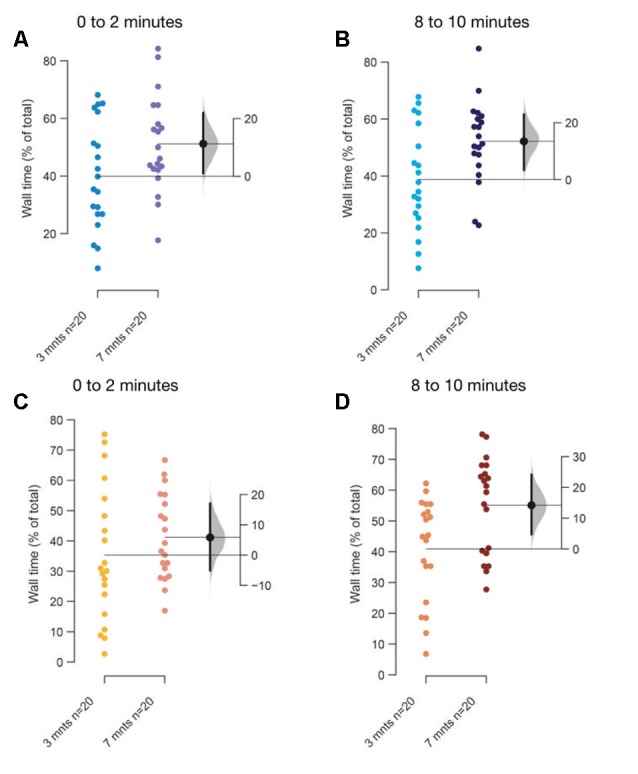
Age-dependent differences in thigmotaxis. The mean difference between 3–5 months and 7–9 months fish when tanks are top lit **(A,B)**, or bottom lit **(C,D)** are shown in Gardner-Altman estimation plots. The percentage of total time along the edges of the tank in **(A)** first 2-min, and in **(B)** last 2-min when tanks are lit from the top. The percentage of total time along the edges of the tank in **(C)** first 2-min, and in the **(D)** last 2-min when tanks are bottom lit. Each dot in the group represents the response of one individual. *N* = 20 per group. Groups are plotted on the left axis, while the mean difference between the groups is depicted as a black dot and is plotted on a floating axis on the right. Ends of the bar around the mean shows 95% confidence interval. The shaded region in gray is 5,000 bootstrap sampling distribution.

**Figure 7:**
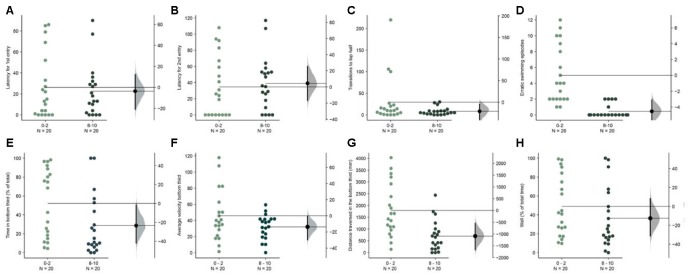
Faster acclimation in wider tank. The mean difference between the first 2 (0–2) and the last 2-min (8–10) are shown in Gardner-Altman estimation plots. The latency to make the **(A)** first entry, **(B)** second entry, to the top half of the tank, the frequency of **(C)** such transitions and **(D)** of the erratic swimming episodes. The **(E)** total percentage of the time, **(F)** average velocity (in mm/s), **(G)** average distance traversed (in mm) in the bottom third of the tank. **(H)** Percentage of time of the total spent swimming along the edge (thigmotaxis). Each dot in the group represents the response of one individual. *N* = 20 per group. Groups are plotted on the left axis, while the mean difference between the groups is depicted as a black dot and is plotted on a floating axis on the right. Ends of the bar around the mean shows 95% confidence interval. The shaded region in gray is 5,000 bootstrap sampling distribution.

**Figure 8:**
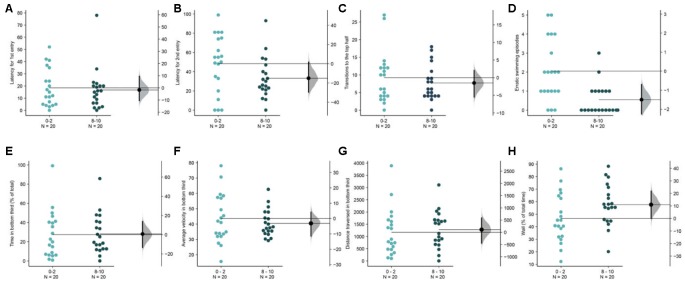
Acclimation in brightly lit novel tanks. The mean difference between the first 2 (0–2) and the last 2-min (8–10) are shown in Gardner-Altman estimation plots. The latency to make the **(A)** first entry, **(B)** second entry, to the top half of the tank, the frequency of **(C)** such transitions and **(D)** of the erratic swimming episodes. The **(E)** total percentage of time, **(F)** average velocity (in mm/s), **(G)** average distance traversed (in mm) in the bottom third of the tank. **(H)** Percentage of time of the total spent swimming along the edge (thigmotaxis). Each dot in the group represents the response of one individual. *N* = 20 per group. Groups are plotted on the left axis, while the mean difference between the groups is depicted as a black dot and is plotted on a floating axis on the right. Ends of the bar around the mean shows 95% confidence interval. The shaded region in gray is 5,000 bootstrap sampling distribution.

**Figure 9:**
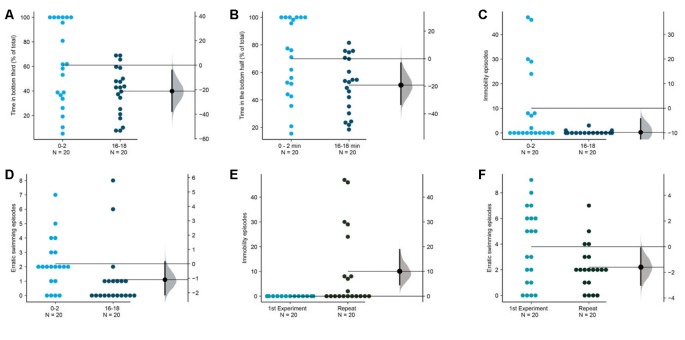
Longer duration improves measures of acclimation. The mean difference between the first 2 (0–2) and the last 2-min (16–18) are shown in Gardner-Altman estimation plots. The percentage of total time in the **(A)** bottom third and **(B)** bottom half, the frequency of **(C)** immobility episodes and **(D)** erratic swimming episodes are reduced in the last 2-min. Fish in this repetition show more **(E)** immobility episodes and fewer **(F)** erratic swimming episodes in the first 2-min compared to the fish in the first experiment shown in Figure 1. Each dot in the group represents the response of one individual. *N* = 20 per group. Groups are plotted on the left axis, while the mean difference between the groups is depicted as a black dot and is plotted on a floating axis on the right. Ends of the bar around the mean shows 95% confidence interval. The shaded region in gray is 5,000 bootstrap sampling distribution.

Males and females showed a similar initial response to the novel tank for most measures, except average velocity (Supplementary Figure S1). A comparison between males and females during the first 2-min of the assay shows that the only notable difference is that males swim at a higher velocity than females [mean difference = 27.5 mm/s (95 CI 38.8, 12.3), *p* = 0.0008]. This difference persisted throughout the duration of the assay (data not shown).

### Acclimation Is Difficult to Observe When Tanks Are Illuminated From the Bottom

Next, we examined age-matched adult siblings of the fish used in the assay above in the same novel tank, but this time the tanks were illuminated from beneath. Among the parameters that showed an acclimation related change above, only a reduction in erratic swimming or darting in the last 2-min compared to the first 2-min was notable [Figure 3D; mean difference = −3.3 (95 CI −4.5, −1.85), *p* = 0.0001]. All the other parameters directly dependent on the initial diving response—time in the bottom third of the tank, latency to enter the top half of the tank, the number of such transitions—show a trend similar to tanks illuminated from the top, but the decrease was marginal and the effects small (Figures 3A–C,E–H).

The main reason for marginal decrease appears to be a stunted diving response initially when tanks are illuminated from the bottom rather than an inability to acclimate. This is evidenced by the observation that in the first 2-min (Figures 4A,B) fish spend less time at the bottom of the tank and consequently have fewer transitions to the top half of the tank (*p* = 0.02 and 0.04, respectively), when tanks are illuminated from the bottom compared to from the top. This marginal initial effect becomes more pronounced over 10 min as fish in bottom lit tanks continue to swim in the top two-thirds of the tank (Figures 4C,D; *p* = 0.003 and 0.007, respectively). Therefore, though the endpoints used to measure anxiety and anxiolytic effects change in the expected direction when tanks are illuminated from beneath, the magnitude of the change is smaller due to a stunted diving response at the start of the assay.

### Older Adults Acclimate Faster and Are Less Anxious in Novel Tanks

In the next experiment, we examined if the response of the fish is consistent across age. We examined 7–9 months fish as many experiments have previously reported using fish between 3–12 months old, which is quite a wide range. One set of fish was examined in each of the two conditions of illumination described above.

Older fish are less sensitive to the illumination from the bottom. They dive initially and recover by the end of the assay duration (Figures 5A–D). This is apparent due to the decrease in latency to enter the top the first time [mean difference = −29.2 s (95 CI −41.2, −10.6), *p* = 0.003], the second time [mean difference = −29.9 s (95 CI −41.6, −12.1), *p* = 0.002], and the total number of such transitions [mean difference = −19.6 (95 CI −36.5, −7.25), *p* = 0.017]. Additionally, erratic swimming episodes decreased significantly in the last 2-min compared to the initial 2-min [Figure 5D; mean difference = −5.15 (95 CI −6.35, −4.05), *p* < 0.0001].

As seen for younger fish in the first experiment, when the tanks are illuminated from the top transitions [mean difference = −20.1 (95 CI −30.6; −10.2), *p* = 0.003] and erratic swimming [mean difference = −4.15 (95 CI −5.47; −3.15), *p* = 0.0002] showed a change in the same manner in older adults. Latency for the first [Figure 5E; mean difference = −5.6 s (95 CI −16.3, 2.5), *p* = 0.2] and second entry [mean difference = −15.5 s (95 CI −31.3, −1.75), *p* = 0.05] to the top half also change in the same direction as experiment 1, but these effects are marginal, once again suggesting faster recovery among older adults.

A major change in the swimming behavior with the age appears to be that older fish prefer to swim along the walls and make fewer transitions through the center of the tank (thigmotaxis), compared to younger fish as they acclimate irrespective of the illumination conditions (top lit Figures 6A,B; bottom lit Figures 6C,D). Unexpectedly therefore, thigmotaxis appears to increase in older fish towards the last 2-min (Figures 6B,D) compared to the first 2-min in the assay (Figures 6A,C).

Having established that the two most important parameters that vary across studies influence the results obtained from a novel tank assay, we next performed three more exploratory experiments.

### Fish Acclimate Faster in Wider Tanks

The dimensions of our novel tank were based on the size of the tank used in previous studies which were narrow and range between 5–7 cm in width (for example, in Levin et al., 2007; Egan et al., 2009). As only two-dimensional videos are required, the standard aquaria tanks used in these studies are adequately suited for the need. In the next experiment, we asked if fish acclimate better or worse in novel tanks that were twice as wide (width = 14 cm). We found that several parameters that show a trend in the first experiment (Figures 2E–G) now showed statistically significant differences when the first 2-min were compared to the last 2-min in the 10-min assay. Fish spent less time [Figure 7E; mean difference = −23.4 s (95 CI −42.8, −2.2), *p* = 0.004], swam slower [Figure 7F; mean difference = −13.6 mm/s (95 CI −30.5, −0.65), *p* = 0.03], and traversed less distance [Figure 7G; mean difference = −1087.4 mm (95 CI −1667.9, −549.55), *p* = 0.0002] in the bottom third of the tank. Erratic swimming episodes (Figure 7D) also changed in the same manner as observed in experiment 1 (Figure 2D). However, latency for first, second entry, and the number of transition to the top half of the tank show only a marginal difference (Figures 7A–C). Further examination reveals that this is likely explained again by a faster rate of acclimation in the wider tank. Compared to the mean value of 34.4 s for the latency to enter the top half the first time in the initial 2-min (Figure 2A), the mean value for the same when the tank is wider is only 27.2 s. Therefore, fish acclimate faster in wider novel tanks.

### Acclimation Is Difficult to Observe in Brightly Lit Novel Tanks

In the experiments described untill until now, the top illumination settings delivered a uniform illumination of 1.5 μW/mm^2^. In the next exploratory experiment, we doubled the intensity of this illumination to 3 μW/mm^2^. Similar to the second experiment (Figure 3) with bottom illumination, it was not possible to detect an acclimation dependent change across most parameters (Figures 8A–H), except for a reduction in erratic swimming or darting in the last 2-min compared to the first 2-min [Figure 8D, mean difference = −1.5 (95 CI −2.35, −0.8), *p* = 0.0008]. Once again, the lack of significant changes in other parameters (Figures 8A–C,E–H) at the end of the assay compared to the beginning can be explained by a stunted initial diving response (Supplementary Figure S2). Compared to the fish in the lower intensity top light in the first experiment, fish in the high intensity lighted tanks spend less time initially [mean difference = −16.8 s (95 CI −29.7, −1.8), *p* = 0.02] in the bottom third. As subjects in this condition are mostly swimming in the middle of the tank, they also make fewer transitions to the top half in the first 2-min [mean difference = −37.7 s (95 CI −53.35, −27.65), *p* = 1.786 e-6 or 0.000001]. Therefore, high-intensity top illumination makes an examination of the novel tank induced anxiety and recovery difficult to quantify.

### Longer Duration for Acclimation

Finally, as the last exploratory experiment, we asked if the measures that showed a trend of decrease in the first experiment (Figures 2E–G) reach statistical significance if acclimated for a longer duration. To address this question, we repeated the first experiment with the same lighting conditions (low intensity of 1.5 μW/mm^2^ from the top) but observed the subjects for 18 min instead of 10 min. This duration is thrice as long as the duration normally used (Levin et al., 2007; Egan et al., 2009). We also analyzed the time in the bottom half rather than a third of the tank as a few studies report this instead. We found that indeed fish show changes suggestive of improved acclimation as they spend less time in the bottom third [mean difference = −21.1 (95 CI −37.3, −3.6), *p* = 0.020] or in the bottom half [mean difference = −19.2 (95 CI −33.2, −2.9), *p* = 0.018] when their behavior between 0–2 min is compared with their behavior between 16–18 min (Figures 9A,B). Apart from a reduction in the erratic swimming episodes like the first experiment (Figure 9C), we also observed a reduction in immobility episodes in this repetition [Figure 9D; mean difference = −9.8 s (95 CI −18.5, −4.3), *p* = 0.010]. Further analysis comparing fish in experiment 1 with this dataset showed that fish in the repetition displayed more episodes of immobility [Figure 9E, mean difference = −10.05 episodes (95 CI 4.6, 18.8), *p* = 0.0007] and fewer erratic swimming episodes in the first 2-min [Figure 9F, mean difference = −1.6 episodes (95 CI −0.1, −3), *p* = 0.04]. Therefore, zebrafish acclimate better to novel tanks in approximately 15 min, and express anxiety in the novel tank initially either by becoming immobile, or by swimming erratically.

## Discussion

Modeling phenotypes associated with human neuropsychiatric disorders in animals is essential to gain a mechanistic understanding of the molecular and genetic players that influence the phenomenon and to devise intervention strategies (Lim and Mathuru, 2017). Zebrafish are used extensively in both cellular and molecular modeling of diseases (Bourque and Houvras, 2011; Santoriello and Zon, 2012; Ablain and Zon, 2013) as well as in pharmacological studies relying on behavioral assays (Cachat et al., 2010; Stewart et al., 2012, 2015; Norton, 2013; Kalueff, 2017; Bao et al., 2019; Fontana et al., 2019).

Among the multitude of assays used to study anxiety, novel tank assays are easy to perform, informative, and are now validated through a large number of studies (Bencan and Levin, 2008; Bencan et al., 2009; Egan et al., 2009; Cachat et al., 2010, 2013; Grossman et al., 2010; Sackerman et al., 2010; Khor et al., 2011; Maximino et al., 2011, 2013a,b; Parker et al., 2013; Pittman and Ichikawa, 2013; Vignet et al., 2013; Kulkarni et al., 2014; Mezzomo et al., 2016; Kalueff, 2017). In this study, we developed a simple pipeline from acquisition to analysis and explored conditions to perform this assay reliably. We expect this will reduce subjective bias in assaying the phenotype, inter-laboratory differences, potential miscommunication about effects observed, and expedite the experimental analysis.

The pipeline used here allows for online tracking of pairs of fish at a time (Figure 1). Using these tools we find that fish acclimate to a novel tank at different rates depending on the conditions of the setup. Among the optimal conditions for this assay is a uniform illumination from above the tank at approximately 1.5 μW/mm^2^. Fish acclimate faster in a wider tank (up to 14 cm), however, a narrow tank (approximately 5–6 cm) used in previous studies to perform this assay is adequate to observe both anxiety and recovery within 10 min. In bottom lit tanks, or when tanks are brightly lit (at 3 μW/mm^2^), the endpoints used to infer anxious behavior in the novel tank, or acclimation to it, show a smaller effect and therefore need to be interpreted with caution. A stunted diving response initially in the first 2-min is the main contributing factor for the smaller effect. Increased brightness in case of high-intensity illumination also increased reflected light from the floor of the tank, likely contributing to the stunted diving. This is consistent with the response of zebrafish in a light/dark novel chamber also used to assess anxiety behavior. In this assay, called scototaxis, given a choice between a white background and a dark one, adult zebrafish prefer spending more time in darker areas and display avoidance of brightly lit lighted areas (Stewart et al., 2011). Taken together with our results, it suggests that adult zebrafish display a negatively phototactic behavior when anxious. The neural circuit mediating such behavior requires further research. Apart from the regular mode of photoreception through the pineal and the eyes, photoreceptivity can occur deep within the brain through photosensitive neurons that express melanopsin (*opn4*) in larval zebrafish that can impact locomotion (Fernandes et al., 2012, 2013). Though this system is also proposed to have a role in fight/flight or freezing behavior (Tay et al., 2011), whether the same is applicable to adult zebrafish is unknown.

Behavioral differences between the sexes have been described in guppies in the past for some exploratory behaviors (Lucon-Xiccato and Dadda, 2016). In each of our experiments, we examined approximately equal number of males and females. We noted some sex differences between male and female swimming patterns consistent with other studies (Tran and Gerlai, 2013; Porseryd et al., 2017), however, we did not observe a reliable or a systematic difference in their rate of acclimation (Supplementary Figure S1).

Finally, our results (Figure 6) suggest that older zebrafish also show increased levels of thigmotaxis regardless of the light conditions when compared to the younger fish over the 10-min recording period. The magnitude of this difference increases over time. This suggests that an increase in thigmotaxis in zebrafish in such settings might be an indicator of anxiolysis. This is counterintuitive in comparison to expectation from rodent studies. One caveat in our experiments was the position of video recording from the front rather than from the top and therefore needs further experimentation before reliable conclusions can be drawn.

**Figure 10:**
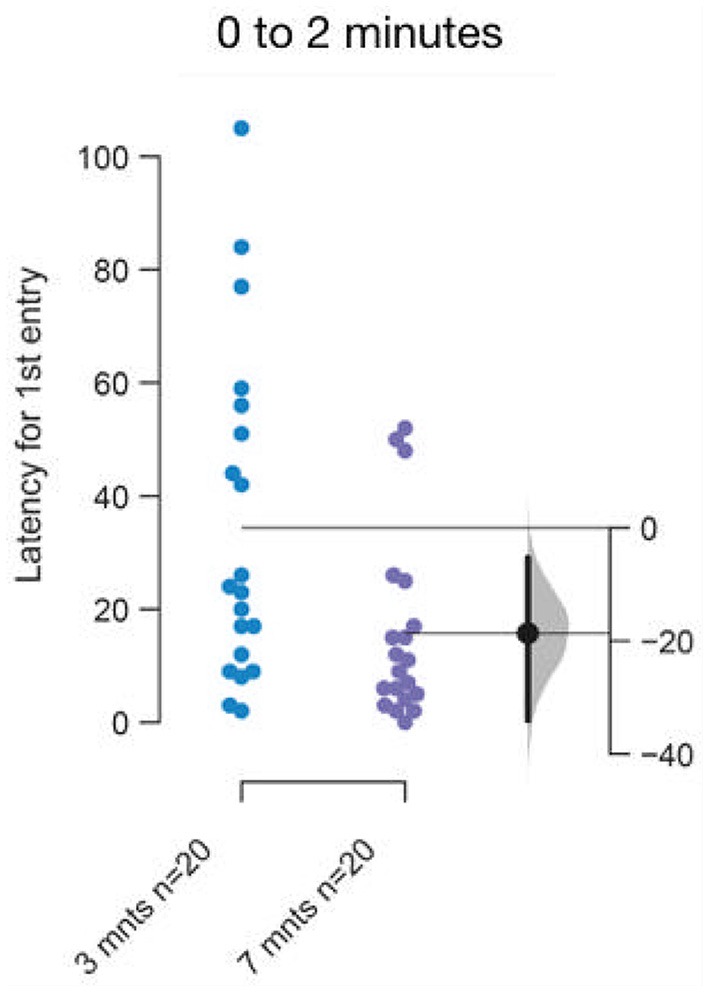
Faster acclimation to novel talk in 7–9 months or older fish. The mean difference between top and bottom lit groups are shown in Gardner-Altman estimation plot. The latency to make the first entry into the top half of the tank is reduced in older fish. Each dot in the group represents the response of one individual. *N* = 20 per group. Groups are plotted on the left axes, while the mean difference between the groups is depicted as a black dot and is plotted on a floating axes on the right. Ends of the bar around the mean shows 95% confidence interval. The shaded region in gray is 5,000 bootstrap sampling distribution.

The difference between the rate of acclimation between young adults, 3–5 months old and 7–9 months or older fish in the ethologically relevant illumination condition was also unanticipated by us [Figure 10; mean difference = −18.65 s (95 CI −34.05, −5.3), *p* = 0.01]. This faster acclimation rate could explain the smaller marginal effect for one out of the four parameters seen in Figure 5. The change in the rate of acclimation is small, yet this result again highlights the need to use age-matched subjects when testing differences between any two conditions.

Several commercial software packages are available to perform these experiments, however, the tools provided with this study require no additional costs or investments, other than the installation of an open source software to execute the Python scripts. They allow the use of any standard web camera to acquire data. Automation also allowed us to streamline the experimental method (please see “Materials and Methods” section) and make efficient use of experimental time. Online tracking avoids the additional time required to track videos after acquisition and the automated analysis of tracked data to quantify the behavioral data reduces dependence on human behavior coders. A complete set of experiments, for example, experiments that require comparing two conditions (a mutant, and a wild type) with *n* = 18–24 per condition, will require a maximum of 4 days of daily experimentation at 3 h/day. These tools are available to the reader as open-source, stand-alone Python scripts. As minimal training in computation is required to use them, researchers with limited experience in coding will be able to utilize them with relative ease. Further, researchers with limited expertise in performing behavioral studies such as those focused on examining developmental defects in genetic mutants in zebrafish, but interested in performing such experiments will also be able to adapt them rapidly.

Based on the results presented above and comparing previous literature (Supplementary Table S1), we recommend that researchers use top light illumination at low intensity (~1.5 μW/mm^2^) and select subjects from a smaller age range (1–2 months). This is particularly relevant when comparing the response of two conditions, such as treated and untreated or mutants and wild type. We expect adopting these recommendations will reduce the noise and increase the “dynamic range” in the study of anxiety and anxiolysis using the zebrafish.

## Data Availability

Publicly available datasets were generated and analyzed in this study. This data can be found here: https://drive.google.com/open?id=1euoh2dVesnXRUQzaFWru54_ENrXOlfzQ.

## Ethics Statement

This study was carried out in accordance with the guidelines recommended by the Institutional Animal Care and Use Committee (IACUC) of the Biological Resource Center at A*STAR. Approved experimental protocols (IACUC 161110) were followed.

## Author Contributions

SH performed experiments and analyzed the data. MK and R-KC developed software and analysis tools. AM conceived and supervised the project, performed experiments and analyzed the data, and wrote the manuscript with assistance from SH.

## Conflict of Interest Statement

The authors declare that the research was conducted in the absence of any commercial or financial relationships that could be construed as a potential conflict of interest.

## Footnotes

^1^http://www.estimationstats.com/#/

## Supplementary Material

FIGURE S1 The first set of experiments examined the response of 3–5 month old fish in top lit tanks. The mean difference between males and females in the first 2 min are shown in Gardner-Altman estimation plots. The latency to make the **(A) **first entry, **(B)** second entry, to the top half of the tank, the frequency of **(C)** such transitions and **(D)** of the erratic swimming episodes. The **(E)** total percentage of time, **(F)** average velocity (in mm/s), **(G)** average distance traversed (in mm) in the bottom third of the tank. **(H)** Percentage of time of the total spent swimming along the edge (thigmotaxis). Each dot in the group represents the response of one individual. N is given under the figure. Groups are plotted on the left axis, while the mean difference between the groups is depicted as a black dot and is plotted on a floating axis on the right. Ends of the bar around the mean shows 95% confidence interval. The shaded region in grey is 5,000 bootstrap sampling distribution. Only notable difference is that males swim at a higher velocity than females [Supplementary Figure S1; mean difference = 27.5 mm/s (95 CI 38.8, 12.3), *p* = 0.0008]. Only notable difference is that males swim at a higher velocity than females [Supplementary Figure S1 F; mean difference = 27.5 mm/s (95 CI 38.8, 12.3), *p* = 0.0008].

FIGURE S2 The mean difference between fish in novel tanks with normal or bright illumination in first 2 min are shown in Gardner-Altman estimation plots. **(A)** Percentage of total time in the bottom third and **(B)** The number of transitions to the top half are plotted show that high-intensity top illumination causes stunted diving response. Each dot in the group represents the response of one individual. *N* = 20. Groups are plotted on the left axis, while the mean difference between the groups is depicted as a black dot and is plotted on a floating axis on the right. Ends of the bar around the mean shows 95% confidence interval. The shaded region in grey is 5,000 bootstrap sampling distribution.

TABLE S1 Results presented here are compared with previous literature.
