# Supplementary material for: An Automated Assay System to Study Novel Tank Induced Anxiety
Source: Front Behav Neurosci. 2019 Aug 8;13:180. doi: 10.3389/fnbeh.2019.00180 (PMC6709859; doi:10.3389/fnbeh.2019.00180)
Supplement: TABLE S1 — Results presented here are compared with previous literature. [file Data_Sheet_1.PDF]

Wild type data extracted from Levin et al., 2007 (minute by minute comparison)	Levin, E. D., Bencan, Z., and Cerutti, D. T. (2007). Anxiolytic effects of nicotine in zebrafish. Physiol. Behav. 90, 54–58. doi:10.1016/j.physbeh.2006.08.026.							
Parameter	Minute	Values (mean ± SEM)		Levin et al., 2007	Top Light 3 months	Top Light 7 months	Bottom Light 3 months	Bottom Light 7 months
**Seconds at bottom**	1	mean	1.34	52.89474	51.58333	34.58333	35.5	37.91667
		upper		58.81474	57.78237	39.93483	42.17554	44.89185
		lower		46.97474	45.3843	29.23184	28.82446	30.94149
	2	mean	1.05	41.44737	33.41667	40.58333	18.83333	29.41667
		upper		49.73737	37.72305	46.55264	22.99403	34.58952
		lower		33.15737	29.11028	34.61402	14.67264	24.24381
	3	mean	0.91	35.92105	34.08333	34.83333	25.25	28.25
		upper		43.42105	38.17739	39.84341	28.59707	31.64951
		lower		28.42105	29.98927	29.82326	21.90293	24.85049
	4	mean	0.8	31.57895	35.33333	29.25	24.91667	25.33333
		upper		39.11895	39.76211	32.45013	27.82228	28.43634
		lower		24.07895	30.90456	26.04987	22.01105	22.23033
	5	mean	0.84	33.15789	36.41667	32.16667	26.08333	31.41667
		upper		40.26789	40.10455	35.71891	29.7969	34.9028
		lower		26.04789	32.72878	28.61442	22.36977	27.93053

Wild type data extracted from Egan et al., 2009	Egan, R. J., Bergner, C. L., Hart, P. C., Cachat, J. M., Canavello, P. R., Elegante, M. F., et al. (2009). Understanding behavioral and physiological phenotypes of stress and anxiety in zebrafish. Behav. Brain Res. 205, 38–44. doi:10.1016/j.bbr.2009.06.022.							
Parameter		Values (mean ± SEM)		Egan et al., 2009	Top Light 3 months	Top Light 7 months	Bottom Light 3 months	Bottom Light 7 months
**Latency to upper half (s)**		mean		27.58621	34.4	26.85	30.95	47.6
		upper		36.2069	40.97	37.72471	38.05836	54.79444
		lower		18.96552	27.83	15.97529	23.84164	40.40556
**Transitions to upper half**		mean		14.25287	132.4	88.2	76.25	89.9
		upper		16.78161	148.41	100.1432	93.43351	106.6015
		lower		11.72414	116.39	76.2568	59.06649	73.19846
**Time in upper half (s)**		mean		77.58621	43.66658	194.5	191.7	186.75
		upper		125.8662	46.52595	207.451	204.6637	198.1501
		lower		29.30621	40.80722	181.549	178.7363	175.3499
**Erratic Movements**		mean		6.436782	11.55	7.7	7.45	9.4
		upper		9.252874	13.17298	8.708125	8.756814	10.66158
		lower		3.62069	9.927015	6.691875	6.143186	8.138422
**Freezing bouts**		mean		0	0.05	2.25	0.35	0.3
		upper		0	0.1	3.047612	0.614326	0.6
		lower		0	0	1.452388	0.085674	0
